# Is patient-reported outcome after treatment of unstable pelvic ring injuries related to pelvic symmetry? A prospective study

**DOI:** 10.1007/s00068-024-02652-2

**Published:** 2024-08-27

**Authors:** Camryn C. Therrien, Kaj ten Duis, Hester Banierink, Jean-Paul P. M. de Vries, Inge H. F. Reininga, Frank F. A. IJpma

**Affiliations:** 1 Department of Trauma Surgery, University of Groningen, University Medical Center Groningen, Groningen, The Netherlands; 2 Department of Surgery, University of Groningen, University Medical Center Groningen, Groningen, The Netherlands

**Keywords:** Pelvic ring injury, Pelvic symmetry, Patient-reported outcome, Functional status, Physical functioning, Quality of life

## Abstract

**Purpose:**

To determine the relation between pelvic symmetry, as measured by the cross-measurement technique, and patient-reported outcome measures (PROMs) in terms of functional status and health-related quality of life.

**Methods:**

In this prospective cohort study, X and Y measurements were taken according to the cross-measurement technique on AP radiographs of patients who were treated for an unstable pelvic ring injury in a level-1 trauma center. Patients completed PROMs at the time of admission (recalled pre-injury score) and one year following their injury, reporting their functional status with the Short Musculoskeletal Function Assessment (SMFA-NL), specifically the lower extremity dysfunction (LED), problems with daily activities (PDA) and mental and emotional problems (MEP) subscales, and quality of life with EuroQol-5D (EQ-5D). Subgroup analyses were also performed. PROMs were used to analyze the relation between pelvic symmetry and patient-reported outcome, using Spearman’s Rank correlation coefficients.

**Results:**

A total of 130 patients (mean age 58 (SD18) years) with an unstable pelvic ring injury were included, of which 95 (73%) sustained type-B injuries and 35 (27%) type-C injuries. Sixty-three (49%) patients were treated operatively. The median pelvic symmetry ratio was 1.01 (IQR: 0.05). Weak or no correlations were found between the pelvic symmetry scores and the outcome measurements (Spearman’s correlation coefficients: LED *r* = 0.09; PDA *r* = 0.11; MEP *r*=-0.02; and EQ-5D *r*=-0.08). Subgroup analyses revealed no correlations.

**Conclusions:**

No significant relation was found between pelvic symmetry, measured radiologically, and functional status and health-related quality of life, one year following an unstable pelvic ring injury.

## Introduction

Traumatic pelvic ring injuries include all major or minor disruptions in the pelvic ring due to trauma [1]. These injuries have major consequences on patients’ quality of life and physical functioning [2–5], especially when displacement and persistent pelvic ring deformity are sustained [6–8]. There are three main classifications of a pelvic ring injury: type-A, which is considered stable, type-B which is defined as rotationally unstable but vertically stable and type-C, which is both rotationally and vertically unstable [9]. Within these classifications, there is a substantial range of severity of the displacement and instability that can occur. There are currently several methods of measuring pelvic displacement radiographically [7, 10–14]; however, there is a lack of clinical evidence supporting the techniques used to measure pelvic symmetry and how they can be used to predict patient outcomes [7, 15].

The cross-measurement technique, which is used to measure pelvic symmetry, involves X and Y measurements from an anterior-posterior (AP) radiograph, that are computed to obtain three symmetry scores:1) a pelvic symmetry value, 2) a deformity index, and 3) a pelvic symmetry ratio [13]. The systematic review by Lefaivre et al. showed that the cross-measurement technique for measuring pelvic symmetry allowed the least observer variability and that it has excellent reliability [13]. Therefore, this technique was used in this study. This technique was explored by Pastor et al. while investigating the correlation between the quality of pelvic reduction and clinical outcome. They concluded that there was a moderate correlation between clinical outcome with the amount of radiographic pelvic reduction (i.e. restoring pelvic symmetry) when measured using this technique [7]. According to Pastor et al., this technique of radiographic measurement has the potential to be a predictor of outcomes following pelvic ring injuries. However, they also concluded that studies with adequate sample sizes are required to further assess this relation [7]. On the other hand, Kukubo et al. showed that poor radiographic outcome did not prove to be a predictor of poor functional outcome after one year for patients with unstable pelvic fractures. Although, in that study, a different measurement technique for pelvic displacement was used [14]. Thus, there is no consensus about the relation between pelvic symmetry and patient-reported functional status and health-related quality of life.

The aim of this study was to determine the relation between pelvic symmetry measured using the cross-measurement technique and patient-reported functional status and health-related quality of life one year after an unstable pelvic ring injury. The question is therefore: is there a relation between pelvic symmetry and patient-reported functional status and health-related quality of life?

## Patients and methods

### Patients

During this prospective longitudinal cohort study, all patients who were over 18 years of age, who survived the trauma, who did not have any known cognitive disorders, who did not have a congenitally, or pre-trauma deformed pelvis, who were able to communicate in the Dutch language, and who were treated for a pelvic ring injury between January 2017 and October 2021 in the University Medical Centre Groningen (UMCG) were informed about the study and asked to participate. The UMCG is a Level-1 trauma center and a secondary and tertiary referral center for the treatment of pelvic injuries in the northern part of the Netherlands. Only patients who had PROMs and radiographs one year following the injury were included in this analysis. Additionally, only patients with type-B or -C injuries were included as those are the injury types that can result in displacement of the pelvic ring and asymmetry [9]. Patient information was prospectively collected and entered into the database upon clinical presentation by reviewing each patient’s medical and surgical records. Patient characteristics that were collected were gender, age, whether it was an isolated pelvic ring injury (no additional fractures other than of the pelvis sustained during the trauma), whether there was an associated acetabulum fracture, high energy trauma (impact of the incident was assumed to be 20 km/h or greater), the mechanism of the injury, whether it was treated surgically and whether operative fixations were placed anteriorly, posteriorly, or both. The treatment decisions were made by the trauma surgeons according to their standard practice. Additionally, initial radiographs were verified by two trauma surgeons and the type of pelvic ring injury was classified according to the AO/OTA Trauma pelvis and acetabulum manual [9]. The local Medical Ethical Review Board reviewed the methods employed and waived further need for approval (METc 2017/543).

### Cross-measurement technique of pelvic ring symmetry

**Fig. 1:**
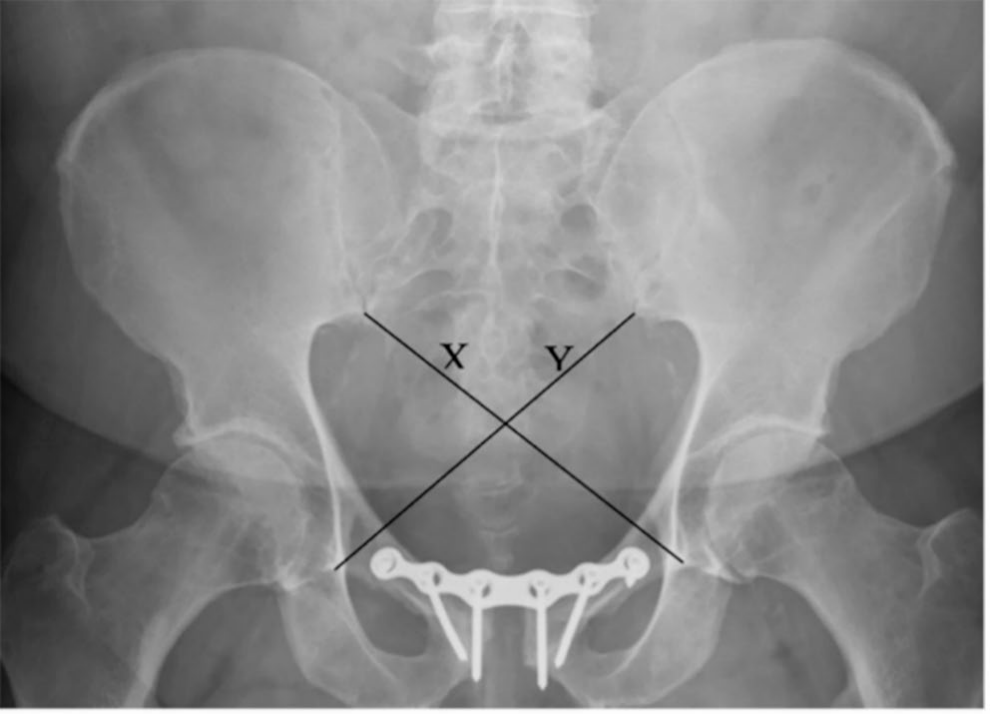
Cross-measurement technique in a patient with a type-B (AO 61B2.3) open book pelvic injury treated with a symphysial plate; (PSV (pelvic symmetry value) = 0.02 cm, DI (deformity index) = 0.00, PSR (pelvic symmetry ratio) = 1.00, deviation from symmetry (DS) = 0) meaning that this is a symmetrical pelvis

**Fig. 2:**
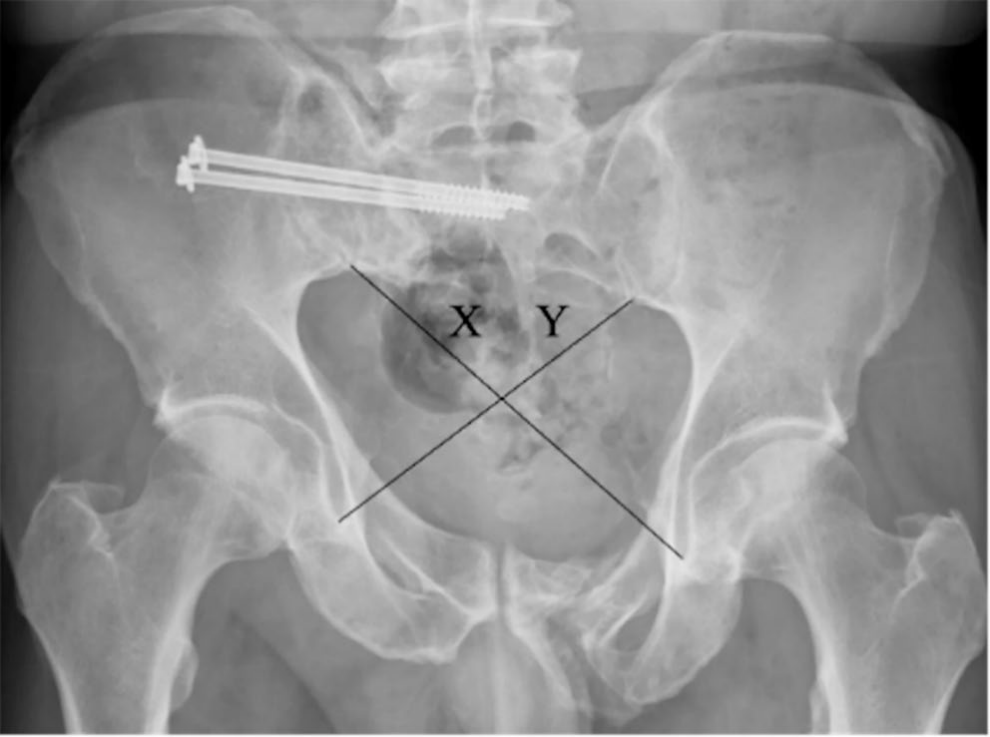
Cross-measurement technique in a patient with a type-C (AO 61C1.3c) vertical shear pelvic injury treated with sacroiliac screws; (PSV = 3.40 cm, DI = 0.10, PSR = 1.23, DS = 0.23) meaning that this is an asymmetrical pelvis

AP radiographs, taken one year after admission, were used for pelvic ring symmetry assessment for all patients. The cross-measurement technique was used to assess pelvic symmetry. This method of radiographic measurement was first described by Keshishyan et al. for pediatric pelvic measurement and was later modified by Lefaivre et al. to be used on adults [10, 13]. It measures the distance from the inferior SI joint (iliac side) to the inferior aspect of the teardrop, the most distal radio-dense area below the acetabulum which resembles a teardrop on an AP film [16]. Measurements are recorded as “X” and “Y” where “Y” is the length from the left SI joint to the right teardrop, and “X” is the opposite of this [13], as shown in Figs. 1 and 2. These measurements were computed in three different ways resulting in values that can be used for analysis. Keshishyan et al. first described its interpretation in two ways. The first is subtracting one measurement from the other, yielding a pelvic asymmetry value (PSV), ABSOLUTE (X – Y), and the second is the deformity index (DI), which takes the film obliquity into account [ABSOLUTE (X – Y)/ (X + Y)]. A third method of interpretation was later added by Lefaivre et al., which was the calculation of the pelvic symmetry ratio (PSR), ABSOLUTE (X/Y) [10, 13].

### Patient-reported outcome measurements (PROMs)

The PROMS were indicated by the patients themselves in terms of functional status and health-related quality of life using PROMs one year following the injury. PROMs from one year after the injury were recorded because it has been suggested by literature that a representative outcome in terms of functional status and health-related quality of life is achieved after 12 months for most patients [17, 18]. Additionally, scores from PROMs administered at the time of admission (recalled pre-injury PROMs) were used as an indication of their condition before the injury.

The Dutch version of the Short Musculoskeletal Function Assessment (SMFA-NL) was used to assess functional status. The SMFA-NL consists of 46 items scored on a 5-point scale, and the following subscales can be further derived from this assessment: upper extremity dysfunction, lower extremity dysfunction, problems with daily activities, and emotional problems, which have demonstrated structural validity [19–21]. Scores from the individual items are summed and transformed, resulting in a score ranging from 0 to 100. A higher level of functional status is represented by a lower score. In this study, the subscales of lower extremity dysfunction (LED), problems with daily activities (PDL), and mental and emotional problems (MEP) scores were used.

The Dutch version of the EuroQol-5D 5 L version (EQ-5D) questionnaire was used to assess the health-related quality of life. The EQ-5D is a brief questionnaire that measures health-related quality of life based on five dimensions of health: mobility, self-care, usual activities, pain/discomfort, and anxiety/depression [22]. Each dimension is answered on a scale of one to five. This results in a utility score on a scale of zero to one, with a higher score representing a higher health-related quality of life. This questionnaire is a valid and reliable outcome measurement in patients who have sustained injuries [23].

### Statistics

Statistical analyses were performed using SPSS. For normally distributed data, means and standard deviations (SD) were presented, and for not normally distributed data, medians, and interquartile ranges (IQR) were used. Frequency was presented as N (% of all patients).

For data analysis purposes, the PSR value was subtracted from 1 so that the absolute values of PSR could be analyzed. With this, 0 represents perfect symmetry and a higher value represents a greater amount of asymmetry. This absolute PSR value was named deviation from symmetry (DS).

Spearman’s Rank correlation coefficients were calculated and were used to assess the association between pelvic symmetry, as measured by the cross-measurement technique and expressed as DS, and the subscales of the SMFA-NL and EQ-5D, presented with a 95% confidence interval (95% CI) and with a significance value of *p* ≤ 0.05 [24]. Spearman’s Rank correlation coefficients were interpreted according to Domholdt et al., with 0.00–0.25 representing little if any correlation; 0.26–0.49 weak correlations; 0.50–0.69 moderate; 0.70–0.89 strong; and 0.90–1.00 very strong correlations [25].

Furthermore, subgroup analyses were conducted within the following subgroups: patients who underwent an operative treatment, those who sustained high-energy traumas, elderly patients (≥ 65 years of age at the time of the trauma), and patients with fragility fractures (≥ 65 years of age at the time of the trauma and a low energy fall as the trauma mechanism). As described above, Spearman’s Rank correlation coefficients were also used within each subgroup to determine if there were associations between pelvic symmetry and the PROMs scores.

## Results

### Patients

During this longitudinal prospective study, there were 130 patients with a pelvic ring injury who reported PROMs one year after the time of injury, see Table 1. Of these patients 62 (48%) were female and the mean age was 58 (SD 17.9) years. Ninety-five (73%) sustained AO/OTA classified type-B injuries and 35 (27%) type-C injuries. Of these injuries, 54 (42)were isolated pelvic ring injuries. Ten patients (8%) sustained an associated acetabulum fracture. Eighty-seven (67%) patients were involved in a high-energy trauma. As a result of the injury, 63 (49%) patients were treated operatively, 14 (11%) with anterior fixation, 23 (18%) with posterior fixation and 27 (21%) with both anterior and posterior fixation Table 2.

**Table 1:** Patient characteristics

Patient characteristics	All patients (*n* = 130)
Female, n (%)	62 (48)
Age at the time of injury, mean (SD)	58 (18)
**Type of injury**	
Type-B, n (%)	95 (73)
Type-C, n (%)	35 (27)
Fragility fractures*, n (%)	28 (22)
Isolated pelvic ring injury, n (%)	54 (42)
Associated acetabulum fracture, n (%)	10 (8)
High energy trauma, n (%)	87 (67)
**Injury Mechanism, n (%)**	
Low energy fall	40 (31)
High energy fall	37 (29)
Compression injury	5 (4)
Crush injury	9 (7)
One-sided car/motorbike accident	11 (9)
Pedestrian vs. car/motorbike	18 (14)
Car/motorbike vs. car	10 (8)
Treated operatively, n (%)	63 (49)
**Location of operative fixation**	
Anterior, n (%)	14 (11)
Posterior, n (%)	23 (18)
Both, n (%)	27 (21)

*Fragility fractures are patients > 65 years with a low-energy fall as the injury mechanism

### Pelvic ring symmetry

The median pelvic symmetry value (PSV) of all patients after healing/treatment was 0.67 cm (IQR: 0.94), the deformity index (DI) was 0.02 (IQR: 0.03), the pelvic symmetry ratio (PSR) was 1.01 (IQR: 0.05) and the deviation from symmetry (DS) was 0.04 (IQR: 0.04).

### Patient-reported outcomes at pre-injury and at one year

**Table 2:** PROMs at pre-injury and at one year

PROMs Measurement, median (IQR)	Results pre-injury (*n* = 130)	Result at one year (*n* = 130)
SMFA-NL LED	0 (5.2)	18.8 (31.0)
SMFA-NL MEP	6.2 (15.7)	21.9 (23.0)
SMFA-NL PDL	1.3 (8.8)	25.0 (35.5)
EQ-5D	1 (0.1)	0.81 (0.17)

Dutch version of the Short Musculoskeletal Function Assessment (SMFA-NL), lower extremity dysfunction (LED), Mental and emotional problems (MEP), problems with daily living (PDL), EuroQol-5D (EQ-5D)

### Correlation between pelvic symmetry and patient-reported outcome

**Fig. 3:**
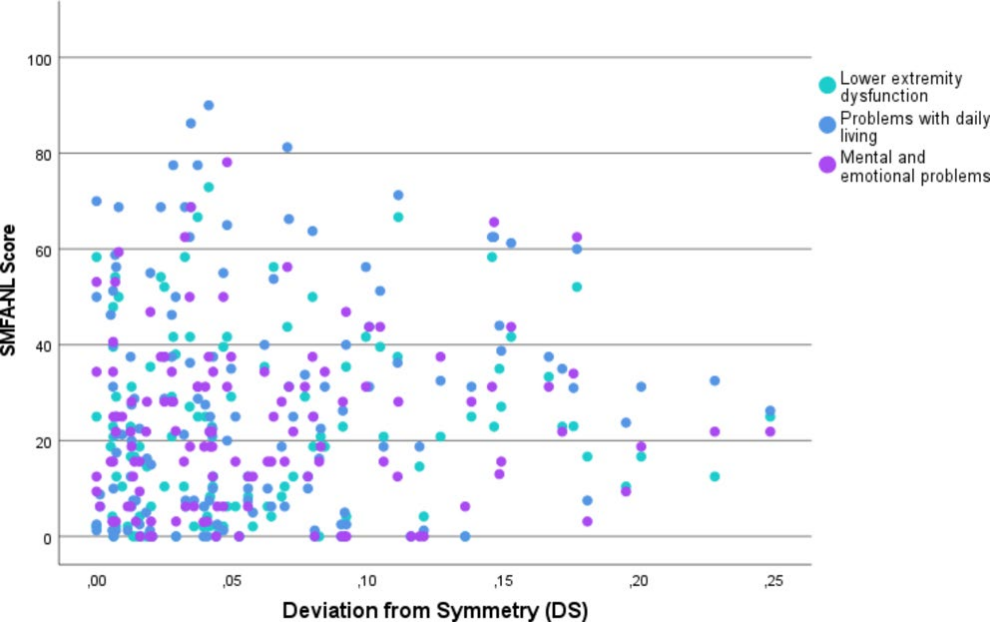
Association between scores on SMFA subscales and pelvic deviation from symmetry (DS). It should be noted that a lower SMFA score represents a higher functional status

**Fig. 4:**
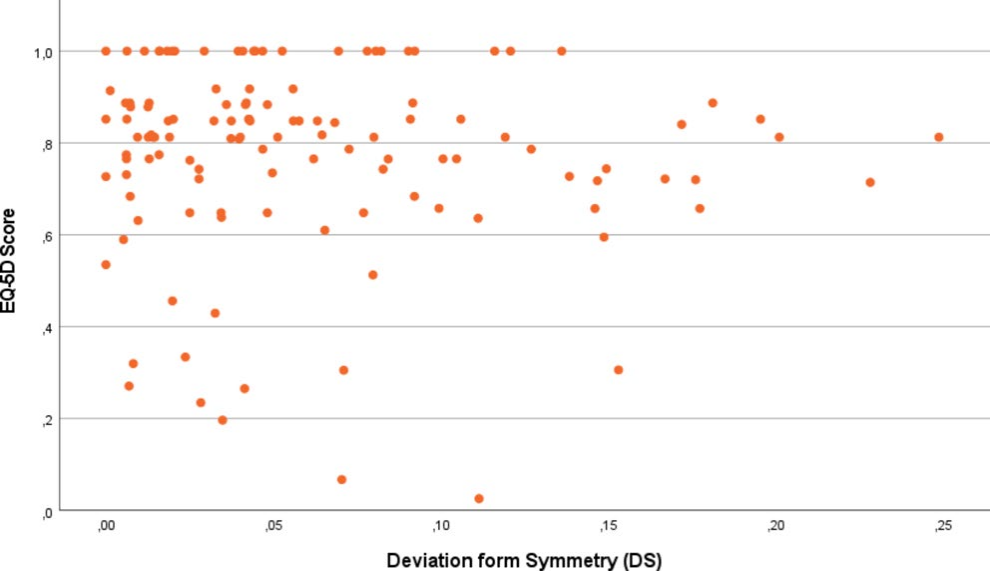
Association between quality of life represented by EQ-5D utility scores and deviation of symmetry (DS)

There were little if any correlations found between the pelvic symmetry scores and the outcome measurements, according to the correlation coefficients benchmarks described by Domholdt et al. Regarding the deviation of symmetry (DS), the correlation with lower extremity dysfunction was *r* = 0.09, *p* = 0.32 (95% CI: -0.09-0.27), with problems with daily living was *r* = 0.11, p-0.25 (95% CI: -0.08-0.28), with mental and emotional problems was *r*=-0.02, *p* = 0.80 (95% CI: -0.16-0.20), and with EQ-5D was *r*=–0.08, *p* = 0.41 (95%CI: -0.25-0.11). These relations are visualized in Figs. 3 and 4.

Furthermore, little if any or weak correlations between pelvic symmetry and patient-reported outcomes were found when analyses were performed within the following subgroups: patients treated operatively (*n* = 44), those who sustained a high-energy trauma (*n* = 80), those over the age of 65 (*n* = 44) and those with a fragility fracture (*n* = 28). The results of these analyses are presented in Table 3.

**Table 3:** Subgroup analyses, the correlation between pelvic symmetry and patient-reported outcome represented by Spearman’s r

	SMFA-NL LED	SMFA-NL MEP	SMFA-NL PDL	EQ-5D
Operative treatment (*n* = 44)				
DS	0.15, *p* = 0.25 (95% CI: -0.12-0.40)	0.22, *p* = 0.08 (95% CI: -0.04-0.46)	0.15, *p* = 0.27 (95% CI: -0.12-0.39)	-0.18, *p* = 0.17 (95% CI: -0.43-0.09)
High energy trauma (*n* = 80)				
DS	0.16, *p* = 0.15 (95% CI: -0.06-0.37)	0.16, *p* = 0.14 (95% CI: -0.06-0.37)	0.19, *p* = 0.09 (95% CI: -0.04-0.39)	-0.13, *p* = 0.24 (95% CI: -0.34-0.09)
> 65 years old (*n* = 49)				
DS	0.12, *p* = 0.43 (95% CI: -0.18-0.40)	-0.04, *p* = 0.79 (95% CI: -0.33-0.26)	0.10, *p* = 0.50 (95% CI: -0.20-0.39)	-0.05, *p* = 0.75 (95% CI: -0.33-0.25)
Fragility fracture (*n* = 28)				
DS	-0.05, *p* = 0.81 (95% CI: -0.43-0.35)	-0.28, *p* = 0.16 (95% CI: -0.60-0.13)	0.02, *p* = 0.92 (95% CI: -0.38-0.41)	0.01, *p* = 0.95 (95% CI: -0.37-0.39)

Dutch version of the Short Musculoskeletal Function Assessment (SMFA-NL), lower extremity dysfunction (LED), Mental and emotional problems (MEP), problems with daily living (PDL), EuroQol-5D (EQ-5D), deviation from symmetry (DS)

### Case examples

**Fig. 5:**
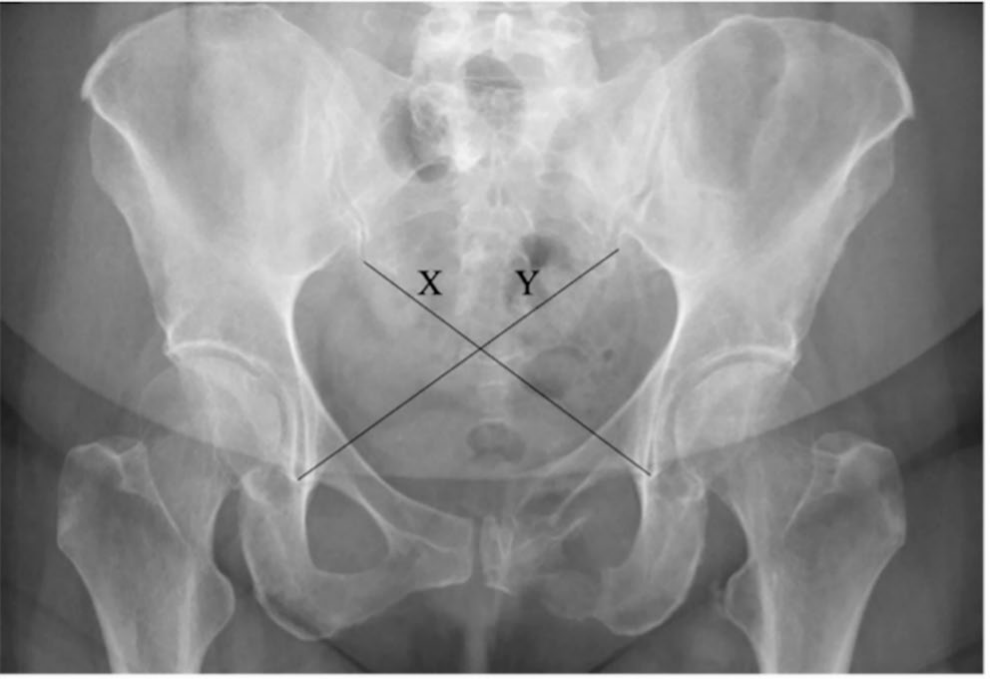
Patient with a type-B (AO 61B2.1a) lateral compression pelvic ring injury treated nonoperatively with some asymmetry and good PROMs. Pelvic *Symmetry*: PSV = 2.01 cm, DI = 0.06, PSR = 0.88, DS = 0.12; Patient-reported outcome: SMFA-NL LED:4.2, PDL:1.3, MEP: 0.0, EQ-5D:1.00

**Fig. 6:**
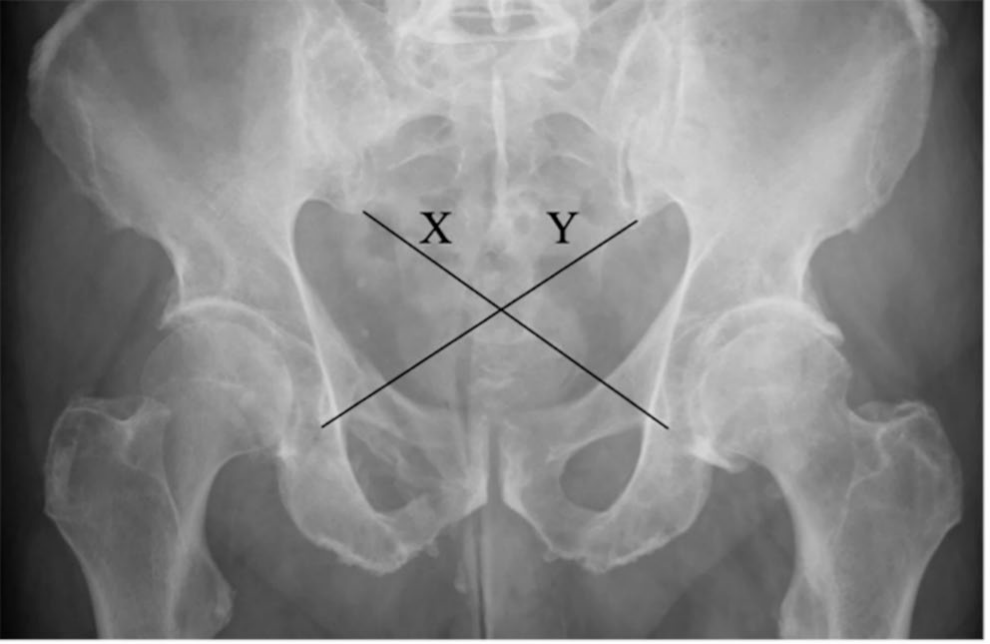
Patient with a type-B (AO 61B2.1a) lateral compression pelvic ring injury treated nonoperatively with symmetrical pelvis and poor PROMs. Pelvic *Symmetry: PSV =* 0.00*cm*,* DI = 0.0*0, *PSR =* 1.00, DS = 0; Patient-reported outcome: LED:58.3, PDL:70.0, MEP:53.1, EQ-5D:0.53

## Discussion

This prospective cohort study investigated the relation between pelvic symmetry measured by the cross-measurement technique and PROMs in terms of functional status and health-related quality of life in patients treated for an unstable pelvic ring injury. Our findings showed weak or no correlations between pelvic symmetry and patient-reported outcomes. Subgroup analyses were additionally performed (e.g. operatively treated patients, those who sustained a high-energy trauma, those over the age of 65, and patients with a fragility fracture), wherein there were also weak, or no correlations found. This shows that there is no direct relation between the pelvic symmetry measured on AP radiographs and patient-reported outcomes in terms of functional status and health-related quality of life. We observed that there were patients who had some pelvic asymmetry with good patient-reported outcomes (Fig. 5), and in contrast, patients who had good pelvic symmetry but poor patient-reported outcomes (Fig. 6). Overall, the clinical relevance of our findings is that anatomic deviations may not necessarily lead to impairments in functional status or health-related quality of life. There are other factors that play a role in the recovery process of a pelvic ring injury. For instance, sociodemographic parameters and pre-injury existing comorbidities and mental health issues are shown to be predictors of post-injury outcomes in terms of functional status [26] and health-related quality of life after traumatic injuries [27]. Especially, emotional coping and resilience are psychological factors that are predictive of better health-related quality of life following injury [28]. Knowing that pelvic symmetry is not a main predictor of a patient’s health-related quality of life and functional status, surgeons should not view perfect pelvic symmetry as a primary goal of treatment and should not choose to treat more aggressively to achieve perfect symmetry.

These findings contradict previous literature by Pastor et al. stating that there is a correlation between symmetry measured by the cross-measurement technique and outcome, and their statement that this measurement technique could be used to predict patient outcome [7]. However, their study included only 31 patients and therefore does not represent a comprehensive picture of potential pelvic deformities. A study that does support our findings investigated potential functional outcome predictors in patients with pelvic ring fractures, conducted by Kokuboo et al. [15]. Their study determined that poor radiological outcome was not a significant outcome predictor one year after the injury [15]; however, they defined radiological outcome simply with a displacement measurement in terms of millimetres and did not use a recognized measurement technique, as we used in our study. Our study adds to previous literature because it is a prospective study with a substantial number of patients using established measurement techniques and valid PROMs.

Some strengths and limitations should be addressed. The prospective study design allows no recall biases for patient-reported outcomes. Moreover, the heterogeneous and substantial size of the sample of this study provides a realistic representation of patients presenting with an unstable pelvic ring injury at a level-1 trauma center. We chose to use the cross-measurement technique, as it has been determined by Lefaivre et al. that it allows the least observer variability and has excellent reliability [13]. A limitation of the study might be that not all AP pelvic radiographs have been made by the same person, on the same X-ray machine, and according to a standardized X-ray protocol as they were made during the standard care of their injuries. It is possible that due to discomfort and pain, some radiographs were obtained from a slightly different angle. However, radiographs were verified by two trauma surgeons and finally, our prospective series represents clinical practice.

## Conclusion

Based on the results of this study, there are weak to no relationships between pelvic symmetry and patient-reported outcomes in terms of functional status and health-related quality of life.

## Data Availability

No datasets were generated or analysed during the current study.
